# Effect of Fusidic Acid on the Kinetics of Molecular Motions During EF-G-Induced Translocation on the Ribosome

**DOI:** 10.1038/s41598-017-10916-8

**Published:** 2017-09-05

**Authors:** Riccardo Belardinelli, Marina V. Rodnina

**Affiliations:** Max Planck Institute for Biophysical Chemistry, Department of Physical Biochemistry, Am Fassberg 11, 37077 Göttingen, Germany

## Abstract

The translocation step of protein synthesis entails binding and dissociation of elongation factor G (EF-G), movements of the two tRNA molecules, and motions of the ribosomal subunits. The translocation step is targeted by many antibiotics. Fusidic acid (FA), an antibiotic that blocks EF-G on the ribosome, may also interfere with some of the ribosome rearrangements, but the exact timing of inhibition remains unclear. To follow in real-time the dynamics of the ribosome–tRNA–EF-G complex, we have developed a fluorescence toolbox which allows us to monitor the key molecular motions during translocation. Here we employed six different fluorescence observables to investigate how FA affects translocation kinetics. We found that FA binds to an early translocation intermediate, but its kinetic effect on tRNA movement is small. FA does not affect the synchronous forward (counterclockwise) movements of the head and body domains of the small ribosomal subunit, but exerts a strong effect on the rates of late translocation events, i.e. backward (clockwise) swiveling of the head domain and the transit of deacylated tRNA through the E′ site, in addition to blocking EF-G dissociation. The use of ensemble kinetics and numerical integration unraveled how the antibiotic targets molecular motions within the ribosome-EF-G complex.

## Introduction

Translation of an mRNA comprises repetitive cycles of decoding, peptide bond formation, and translocation during which the ribosome moves along the mRNA by one codon at every elongation step. The arrival and departure of elongation factor G (EF-G), the movements of the tRNA molecules, and the motions of the ribosomal subunits render the translocation step the most dynamic and complex phase of protein synthesis. The two tRNAs bound to the P and A site spontaneously move from the classical to hybrid orientations upon peptide bond formation^1^. At the same time, the small ribosome subunit (SSU) rotates relative to the large subunit (LSU) from the classical non-rotated (N) to the hybrid rotated (R) state, and the head domain of the SSU swivels relative to the body domain forward in the direction of tRNA movement^2–5^. Also the ribosomal protein L1 changes its position from open to closed where it forms a contact with the P/E tRNA elbow region^6–9^. The current minimal model of translocation entails five steps (see review^10^ and references therein). (1) Binding of EF-G–GTP to the pre-translocation (PRE) complex stabilizes the R state. EF-G can also bind to the ribosome in the N state and accelerates the transition to the R state^11^. (2) GTP hydrolysis by EF-G and a conformational rearrangement of the factor lead to tightening of its interaction with the ribosome. At this step the body domain of the SSU starts to move from the R towards the N state; in contrast, the head domain of the SSU remains in a swiveled state. This leads to the unlocking of the ribosome (step 3), allowing for rapid Pi release from EF-G and tRNA movement from the A and P to the P and E sites on the SSU. The SSU body continues its rotation towards the N state, while the SSU head domain starts swiveling in the backward direction. (4) The E-site tRNA moves into the E′ state and EF-G dissociates from L12, but is probably not completely released from the ribosome. (5) The backward motion of the SSU head and body restores the N state, whereas the deacylated tRNA and EF-G–GDP are released from the post-translocation (POST) state of the ribosome. The five steps describe the kinetic path between the PRE and the POST states through four translocation intermediates^12^. These intermediates differ by the tRNA positions and the conformation of the SSU body and head domains, and are denoted as chimeric (CHI) states^10, 12, 13^.

Translocation is inhibited by a number of antibiotics, which act by distinct mechanisms. Fusidic acid (FA) is a well-known inhibitor of EF-G function. FA does not bind to free EF-G or ribosomes, but targets the ribosome–EF-G complex by binding to an inter-domain pocket close to the GTP-binding site of EF-G and inhibits EF-G turnover by blocking EF-G dissociation^14–16^. In addition, FA may target several other EF-G-containing states, among them an early translocation intermediate, as implied by the inhibition constant for FA binding^17^. However, translocation and GTP hydrolysis by EF-G are not affected by FA^17–20^. Single-molecule fluorescence resonance energy transfer (smFRET) experiments indicated that FA blocks EF-G in a late state after tRNA translocation, prior to the dissociation of the E-site tRNA from the ribosome. This state shows a particular arrangement of the P- and E-site tRNAs manifested in a high FRET value for the tRNA-tRNA dye pair^13^. Another smFRET study using multiple FRET reporters also suggested that FA stabilizes the complex in a POST-like configuration that exhibits a compacted tRNA arrangement and increased distance between the SSU head domain and the LSU proteins L1 and L5^21^. Thus, FA binding may prevent late remodeling events in the EF-G–GDP–ribosome complex. However, the exact effect on each step of translocation is not known.

Among the structures of translocating ribosomes^22^, two structures captured the complexes with two tRNAs, EF-G–GDP and FA, but no other ligands such as additional antibiotics^2, 23^. Interestingly, the two structures are quite different. In one structure, representing a cryo-EM structure of the complex, the SSU head is swiveled by 18.4 degree, the body is rotated by 2.5 degree, and the tRNAs are in CHI states (i.e. ap/P and pe/E) with the anticodons in intermediate positions on the SSU and the CCA-ends fully translocated on the LSU^2^. The second structure obtained by X-ray crystallography presents a non-swiveled and non-rotated ribosome conformation with tRNAs in their classic positions (P and E)^23^. Although cryo-EM and X-ray might capture somewhat different states, these structures can be directly compared to the intermediates identified in kinetic studies^12, 21^.

It is not clear when exactly FA binds to the ribosome–EF-G complex, how it affects the kinetics of elemental steps and the overall translocation trajectory, and how the rotation and swiveling of the body and head, respectively, respond to the FA binding. This prompted us to study the effect of FA on the translocation pathway using our recently developed translational fluorescence toolbox which allows us to determine the rates of all five major steps of translocation (rate constants k_1_, k_-1_, k_2_, k_3_, k_4_ and k_5_)^12, 24^. Furthermore, by calculating intrinsic fluorescence intensities (IFI values) of intermediates and comparing them in the absence and presence of FA, we can monitor whether the conformations of intermediates are altered. We show that FA can bind to EF-G in an early CHI state, but exerts its main action at the late steps 4 and 5 of translocation. The antibiotic blocks the dissociation of tRNA from the E′ site, the completion of the backward swiveling of the SSU, and the release of EF-G. These results provide a comprehensive picture of the mechanism of FA action and reconcile previous results of ensemble and smFRET kinetics and the structural work.

## Results

### Real-time kinetic effect of FA

**Figure 1:**
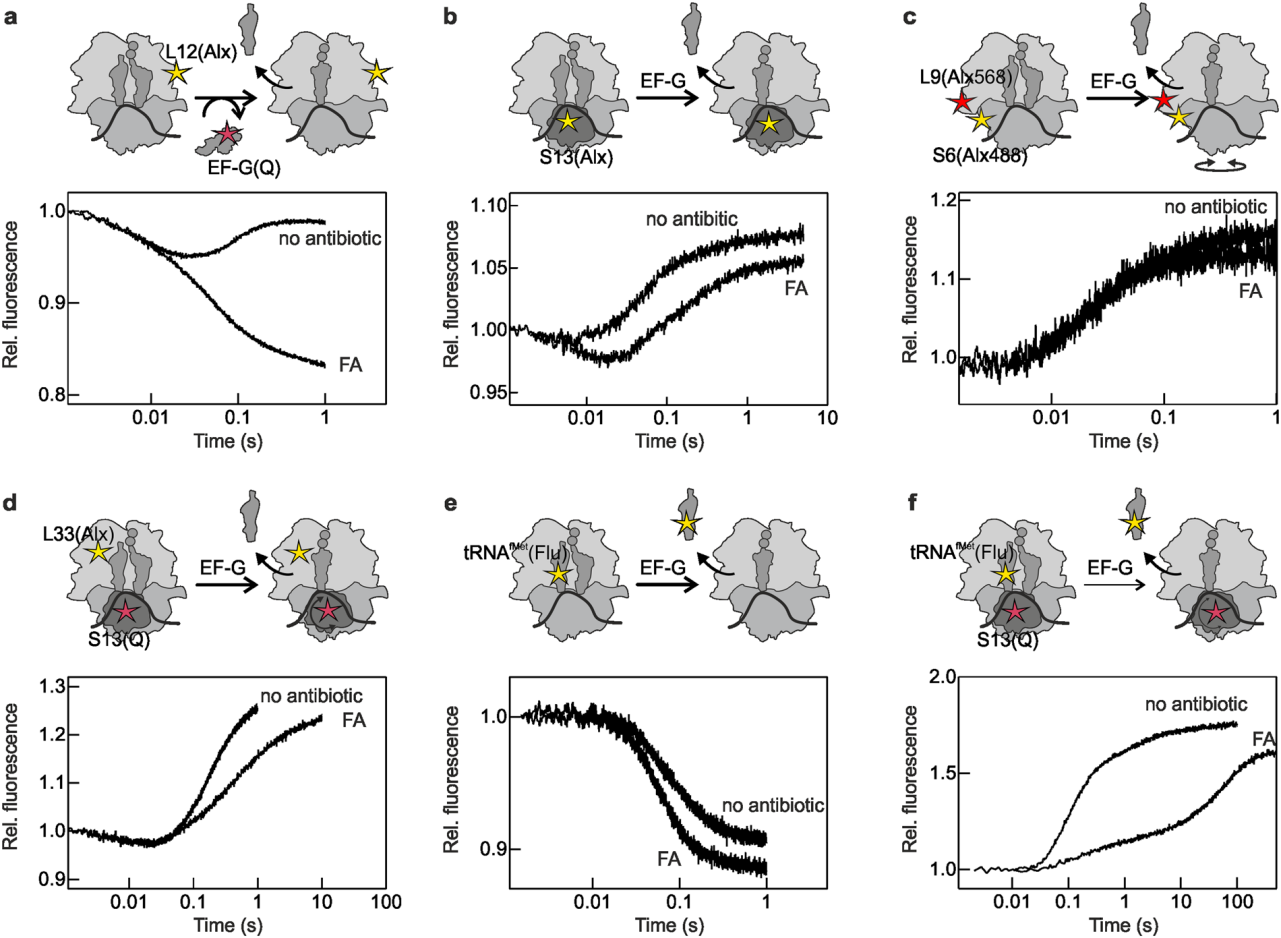
Fluorescence observables and translocation time courses recorded in in the absence (no antibiotic)^12^ or presence of fusidic acid (FA). Schematics of the translocation reaction highlight the positions of the fluorescent reporters. Assignment of fluorescence and FRET changes to distinct translocation steps of taken from^12^. (**a**) FRET between L12-labeled with Alexa 488 and EF-G-labeled with QSY9 used to monitor EF-G binding to and dissociation from the ribosome. (**b**) Fluorescence change of Alexa 488 attached to S13 reflecting EF-G binding and further conformational rearrangements. (**c**) FRET between ribosomal proteins S6 and L9 labeled with Alexa 488 and Alexa 568, respectively, showing SSU body domain rotation relative to the LSU. (**d**) FRET between L33 labeled with Alexa 488 and S13 labeled with Atto540Q reporting on the SSU head domain swiveling. (**e**) Fluorescence change of fluorescein-labeled tRNA^fMet^ used to assess the tRNA movement from the P to the E site. (**f**) FRET between fluorescein attached to tRNA^fMet^ and Atto540Q on S13, which reports on the movement of the P-site tRNA to the E and E′ site and its dissociation from the ribosome.

We used a combination of previously validated fluorescence reporters^11, 12, 24, 25^ to monitor the inhibitory effect of FA (Fig. 1). The selection of reporters allowed us to monitor the binding and dissociation of EF-G measuring L12-EF-G FRET between L12–Alexa 488 and the non-fluorescent acceptor QSY9 on EF-G. EF-G binding is also reflected by a rapid fluorescence change of the SSU protein S13 labeled with Alexa 488; that label also reports on later steps of translocation. The forward and backward motions of the SSU body and head domains were monitored by changes in FRET intensities of Alexa 488–Alexa 568 and Alexa 488–Atto 540Q dye pairs attached to S6-L9 and S13-L33, respectively. The transit of the deacylated tRNA within the ribosome and through the E site was monitored by fluorescence changes of fluorescein attached to tRNA^fMet^ or by FRET between fluorescein and Atto 540Q on the S13-tRNA pair. Purified PRE complex bearing single fluorescence reporters or reporter pairs were mixed with saturating concentration of EF-G–GTP in the presence or absence of FA (200 μM). For each fluorescence-labeled PRE complex, we recorded the time courses of reaction using a stopped-flow apparatus.

In the absence of FA, EF-G recruitment by L12 results in the initial decrease in the fluorescence intensity of the reporter on L12 upon coming in proximity of the quencher on EF-G, followed by a fluorescence recovery when EF-G dissociates from the POST complex (Fig. 1a). When FA is present, we observe the loss of the upward phase indicating that EF-G is not capable to dissociate from the ribosome, as expected from previous reports^2, 14, 15, 21, 23, 25, 26^. From visual inspection of the time courses, the rate of EF-G binding appears very similar in the presence or absence of FA, consistent with the notion that FA does not interfere with EF-G binding (Fig. 1b) ^12, 13, 21^. This is further supported by experiments where the fluorescence change of Alexa 488 attached to protein S13 is monitored. In this case, the rapid downward phase that accompanies EF-G binding was similar in the absence and presence of FA, whereas some differences appeared at later stages, which cannot be dissected based on the S13 signal alone.

We next monitored the effect of FA on the dynamics of the SSU body and head domains (Fig. 1c,d). The time courses of body rotation in the presence and absence of FA were almost completely overlapping, indicating that the body rotation is not affected. The head swiveling in the forward/counter-clockwise direction (which results in the downward phase in FRET time courses) was also independent of the presence of FA. However, the reverse swiveling was markedly slower when FA was present. To follow the transit of the deacylated tRNA from the P to the E site we monitored the fluorescence of tRNA^fMet^, whereas the transit through the E′ site to solution was reported by FRET between labels on tRNA^fMet^ and ribosomal protein S13 (Fig. 1e,f). tRNA^fMet^ fluorescence time courses in the absence and presence of the antibiotic were very similar, suggesting that the movement of the deacylated tRNA^fMet^ from the P to the E site is not hindered by the antibiotic, consistent with a previous kinetic analysis^18^. To monitor the subsequent movements of the E-site tRNA, we used the S13–tRNA^fMet^ FRET pair that reports on the movement of the tRNA from the E to E′ site and the dissociation from the ribosome^12, 25^. In the presence of FA, the movement to the E′ state (the FRET change observed at 0.1–1 s) is slow and the dissociation (seen as FRET change between 20 and 200 s) is even slower than in the absence of the antibiotic (Fig. 1f). This indicates that FA induces a longer retention of the deacylated tRNA^fMet^ in the E′ site prior to being released from the POST complex into solution. Thus, the most pronounced effect of FA is on the backward swiveling of the SSU head domain and on the dissociation of the tRNA from the E′ state, in addition to blocking EF-G dissociation from the ribosome.

### Rate constants of elemental steps of translocation

**Figure 2:**
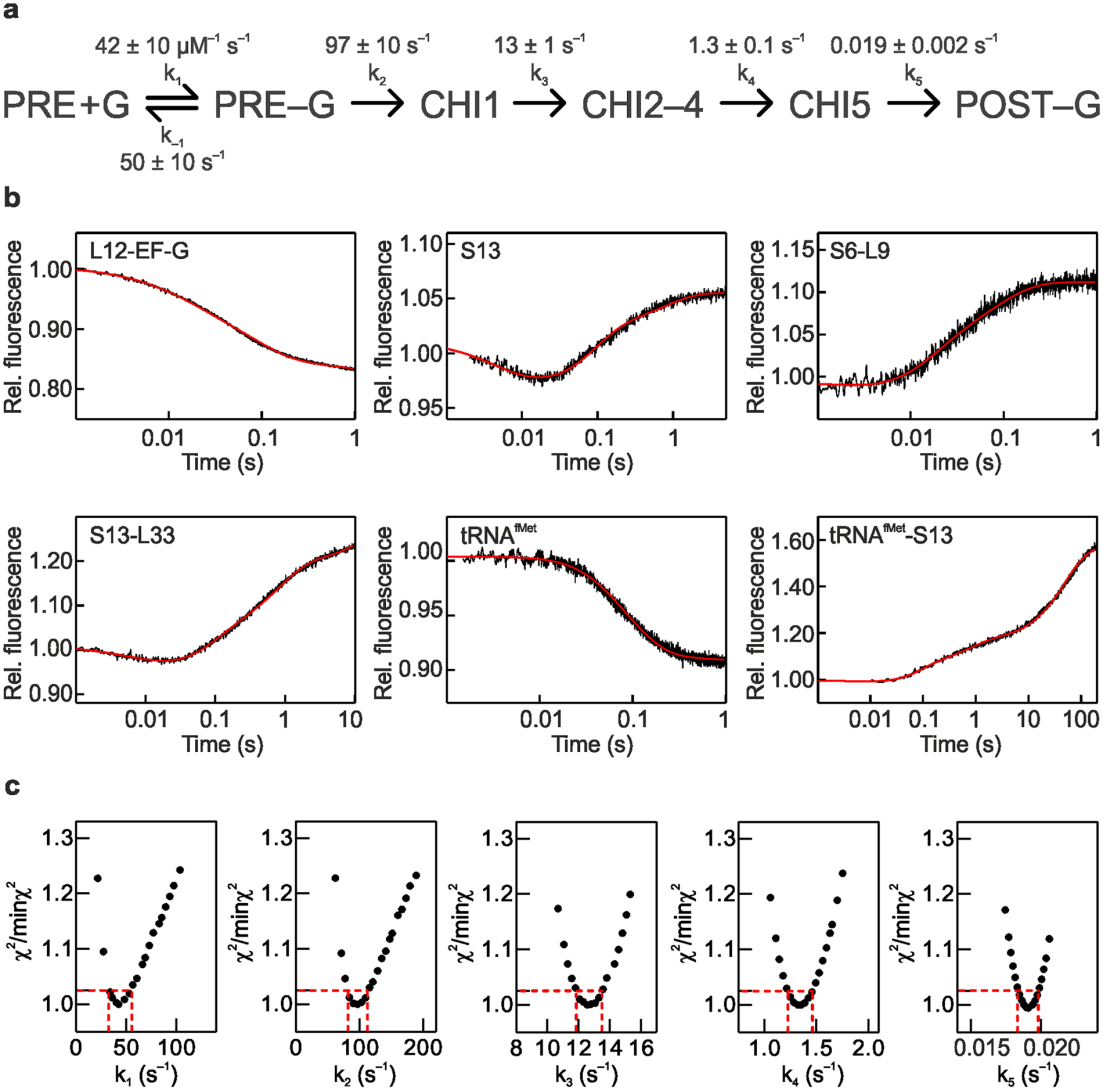
Global analysis of translocation in the presence of FA. (**a**) Schematic of the 5-steps minimum model used for fitting. In step 1 (rate constants k_1_ and k_-1_), PRE complex binds EF-G (G) to form the PRE–EF-G complex which then undergoes several rearrangements leading to translocation and formation of the POST complex. Translocation intermediates, or chimeric (CHI) states, are adopted from previous structural work, ensemble kinetics and single molecule FRET experiments (reviewed in ref. 10). In step 2 (k_2_), EF-G hydrolyzes GTP, engages in translocation, and uncouples SSU head and body movements, resulting in formation of an intermediate CHI1. This allows for the ribosome unlocking in step 3 (k_3_), forming state CHI2. The following states CHI3 and CHI4 entail rapid Pi release from EF-G and stepwise tRNA translocation from the A to P and P to E sites. CHI3 and CHI4 have been characterized by cryo-EM and smFRET methods using specifically stalled complexes, but are not accumulating during unperturbed translocation and are therefore not observed as independent steps in ensemble kinetics; here formation of intermediated CHI2 to CHI4 is grouped into a single kinetic step. In step 4 (k_4_), the tRNA that has been displaced from the P to the E site moves further through the ribosome via at least one distinct intermediate state (CHI5), and finally dissociates from the ribosome (k_5_). Because EF-G does not dissociate in the presence of FA, the final state of the complex is POST–EF-G. (**b**) Translocation time-courses in the presence of FA (black) and the respective fits from numerical integration analysis (red). (**c**) Goodness of the fit as evaluated by KinTek Explorer FitSpace analysis. Dashed red lines reflect the lower and upper boundaries as calculated for a χ^2^ threshold of 1.025 which reflects an estimate of the 95% confidence intervals of the elemental rates (see Manufacturer’s manual and ref. 35).

To elucidate the effect of FA on the kinetics of each translocation step, we used a previously validated 5-steps kinetic model and performed a global fit of all the time courses (Fig. 2). Because FA targets EF-G while bound to the ribosome, rather than free EF-G, and because the initial steps of translocation were not altered (Fig. 1a–c) we assumed that the affinity of EF-G for the PRE complex is unaffected. In the calculations we therefore constrained the K_d_ value, or k_-1_/k_1_, to 65/55 μM calculated from the rate constants for the reaction in the absence of FA^10, 12^, but allowing the values of the binding and dissociation rate constants to adjust freely when numerical integration was searching for the best fitting. Visual inspection of the fits (Fig. 2b) and statistical analysis (Fig. 2c) confirmed the goodness of the fit. The resulting k_1_ = 42 ± 10 μM^−1^ s^−1^ in the presence of FA is close to that determined from multiple titration experiment in the absence of the antibiotic (55 ± 6 μM^−1^ s^−1^ ^12^). The rate of the second step – representing the rearrangements induced by GTP hydrolysis – was also very similar in the absence and presence of FA, 85 ± 10 s^−1^ and 97 ± 10 s^−1^, respectively. The unlocking step, resulting in movement of the tRNAs from the A to P and P to E sites, was 3-times slower (13 ± 1 s^−1^, compared to 43 ± 2 s^−1^; ref. 12). However, the largest effect was observed on the rate of steps 4 and 5, which are 10 and 200 times, respectively, slower than for the reactions in the absence of FA (k_4_ = 1.3 ± 0.1 s^−1^ and k_5_ = 0.019 ± 0.002 s^−1^). Notably, the k_5_ value obtained in the presence of FA pertains to tRNA dissociation only, whereas EF-G remains bound to the ribosome; in contrast, in the absence of the antibiotic, k_5_ reflects the rate constant of dissociation of both tRNA and EF-G.

### Translocation trajectory deduced from the IFI values

**Figure 3:**
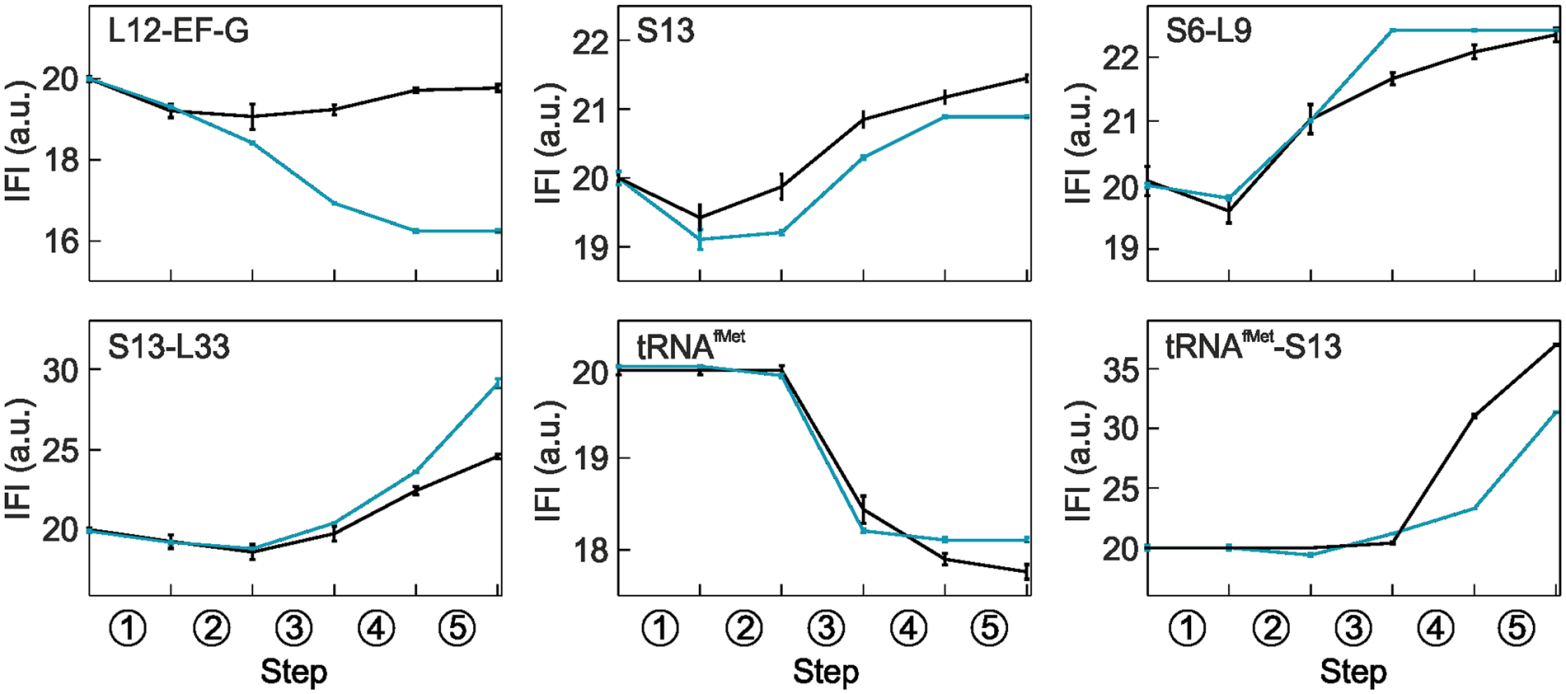
Intrinsic Fluorescence Intensities (IFI) values of the translocation intermediates. IFIs of intermediates during unperturbed translocation with EF-G–GTP (black; ref. 12) compared to IFIs obtained in the presence of FA (light-blue; this work).

The analysis of the intrinsic fluorescence intensities (IFI values) (Fig. 3) and the comparison with the values obtained in the absence of the FA shows which conformational changes and translocation intermediates are most affected by FA. Despite the kinetic differences, the conformations reported by S6-L9 FRET and the tRNA^fMet^ fluorescence are not changed by the presence of FA, suggesting that FA does not alter the translocation pathway. Also the IFI values of the S13 label show the same tendency in the absence and presence of FA; the calculated differences in the exact values are too small to be taken with certainty. Also the calculated difference in the IFI values for the final state reported by the S13-L33 label is somewhat ambiguous, as the states are not identical, because in the presence of FA EF-G remains bound to the ribosome after step 5. The largest deviation from the unperturbed path is observed for L12–EF-G, where the IFI values dramatically changes after step 3, indicating that FA stalls EF-G in a conformation that is not sampled during unperturbed translocation. In the presence of FA, the IFI value of the S13-tRNA^fMet^ pair remains low in step 4 (Fig. 3), which is also clearly seen in the original time courses (Fig. 1f). This indicates that despite the largely unaffected translocation from P to E site, the trajectory of further tRNA movements through the E′ site is changed by FA, that is, it likely does not represent the same intermediate as during the unperturbed translocation.

## Discussion

**Figure 4:**
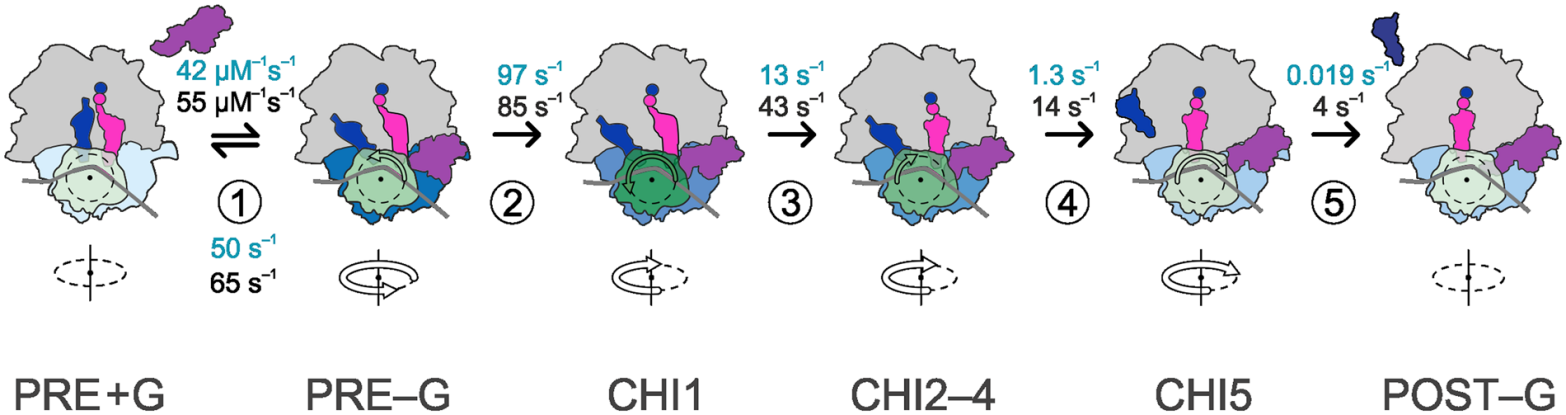
Schematic of kinetic trapping of translocation by FA. The elemental rate constants of unperturbed translocation (black) compared to FA-impaired translocation (light blue). Step①, EF-G binding. Step②, EF-G engagement and uncoupling of the movements of the SSU head and body domains. Step③, ribosome unlocking, which allows for rapid synchronized tRNA movement from the A to P and P to E sites and Pi release from EF-G. Step④, movement of the E-site tRNA to the E′ state. Step⑤, dissociation of EF-G and tRNA leading to formation of the POST complex. The degree of the SSU head domain rotation is indicated by different shades of green, with dark green in the CHI1 state representing the maximum rotation. The degree of SSU body rotation relative to the LSU is shown in different shades of blue, with dark blue in the PRE–EF-G state displaying the maximum degree of body rotation.

Our data provide a comprehensive picture of how FA affects the dynamics of ribosome–EF-G interactions during translocation (Fig. 4). Step 1, i.e. EF-G binding to the ribosome, is not affected, in agreement with previous reports^14, 17, 18, 21^. Step 2 comprises GTP hydrolysis and the ensuing EF-G rearrangements. After hydrolysis, EF-G remains in the EF-G–GDP–Pi form^27^. The rate of the next step 3, which includes ribosome unlocking, translocation of tRNAs from A to P and P to E sites, and Pi release from EF-G, is reduced by a factor of 3 in the presence of FA. This suggests that FA must bind to an early translocation intermediate, consistent with the recent inhibition study^17^. However, because FA binds next to the Pi-binding pocket and may clash with the closed conformation of switch I of EF-G^23^, and given the potential overlap of the γ-phosphate of the nucleotide (or the Pi) with the drug^21, 26^, the most probable scenario is that FA binds to EF-G after Pi is released, a reaction which takes place immediately after the unlocking of the ribosome, but before the tRNAs have moved^27^. The SSU body rotation relative to the LSU is not affected, in agreement with previous reports^28^. With our S6-L9 FRET pair we do not observe the formation of a FA-stalled intermediate with the SSU body that is partially rotated, such as suggested in a recent smFRET study^21^. Most likely, the S13-L1 FRET pair used in that work represents the dynamics of L1 motions, consistent with the effect of FA on the tRNA dissociation from the Eʹ site (see below).

The main inhibitory effect of FA is observed at steps 4 and 5, which represent the movement of tRNA through the E′ site, reverse swiveling of the SSU head domain and the dissociation of EF-G, which are slowed down by FA by 10- and 200-fold, respectively. While EF-G release is blocked by FA, the release of the tRNA from the E′ site may be reversible: tRNA dissociation would favor the formation of the POST–EF-G state, whereas re-binding of tRNA would shift the equilibrium towards the CHI states. The experimental conditions may define which intermediate is captured in structural studies, a CHI state with the significant degree of SSU head swiveling and both E-site tRNA and EF-G bound^2^, or a subsequent POST-like state were the backward swiveling is almost completed^23^. The retention of the E′-site tRNA in the presence of FA is in agreement with previous smFRET studies^13, 21^. Furthermore, our notion that the orientation of the E′-tRNA differs from that in the complex without FA is also corroborated by smFRET studies, which show that the P- and E-site tRNAs are located in a closer (compressed) arrangement^13, 21^. This also provides an explanation why the S13–L1 pair shows an alternative orientation in the FA-stalled complex, as L1 is probably constrained by interactions with the (displaced) E′-tRNA.

The inhibition of EF-G dissociation is the strongest effect of FA. At the concentration of FA used here we do not observe release of EF-G from the POST complex. In contrast, slow EF-G release was observed when GTP was replaced with a non-hydrolyzable GTP analog, GTPγS, and the IFI values of the intermediates formed by steps 4 and 5 changed differently with the nonhydrolyzable analog than with FA^12, 25^. This indicates that the FA-perturbed POST complex represents a structurally different intermediate with respect to complexes stalled with nucleotide analog alone. Earlier studies with mant-GTP/GDP suggested that FA induced a characteristic conformation of EF-G which resulted in an extremely high FRET between a Trp residue in the G domain and the mant group, which did not accumulate when the reaction was carried out without FA^25^. This supports the notion that the FA-stalled conformation of EF-G may be off the main translocation pathway and calls for caution in the interpretation of structural data obtained with FA, in particular with respect to the details of interactions at the nucleotide binding pocket as compared to intermediates of normal translocation. In summary, this work shows that FA binds already at a relatively early step of translocation, probably immediately after Pi release, but inhibits later steps, including dissociation of the E′-tRNA and the reverse swiveling of the SSU head domain, leaving EF-G irreversibly blocked on the ribosome in a unique stalled conformation. This view integrates recent ensemble kinetics, smFRET experiments, and existing cryo-EM structures of FA-stalled functional complexes and provides a consistent picture of how FA affects translocation.

## Methods

### Ribosomes, mRNAs, tRNAs and translation factors

*E. coli* 70S ribosomes and ribosomal subunits, f[^3^H]Met-tRNA^fMet^, f[^3^H]Met-tRNA^fMet^(Flu), [^14^C]Phe-tRNA^Phe^, initiation factors, EF-Tu, and EF-G were prepared as described^12, 25, 27, 29^. mRNA constructs were synthesized by IBA (Göttingen, Germany) using the sequence 5′-GUUAACAGGUAUACAUACUAUGUUUGUUAUUAC-3′^25, 30^. Labeling of ribosomal subunits was carried out essentially as described previously^11, 12, 24, 25, 31–33^. Unless otherwise specified, all experiments were carried out in buffer A (50 mM Tris-HCl (pH = 7.5), 70 mM NH_4_Cl, 30 mM KCl and 7 mM MgCl_2_).

### Ribosome complexes

Preparation and purification of IC and PRE complexes were carried out essentially as described^12, 25, 27, 29^. Briefly, activated 30 S ribosomal subunits (0.5 µM) were mixed with a 1.5-fold excess of 50 S subunits in the presence of f[^3^H]Met-tRNA^fMet^ (2-fold excess), mRNA (3-fold excess), initiation factors IF1, IF2, and IF3 (1.5-fold excess each) in buffer A supplemented with 1 mM GTP. The mixture was incubated for 30 min at 37 °C and the ICs were purified through a 1.1 M sucrose cushion in buffer A. PRE complexes were assembled by mixing equal volumes of IC (see above) and ternary complex (TC), which was prepared as follows. EF-Tu (2-fold excess over Phe-tRNA^Phe^) was incubated for 15 min at 37 °C in buffer A supplemented with 1 mM GTP, 2 mM dithiothreitol (DTT), 3 mM phosphoenolpyruvate (PEP), and 0.5 mg/ml pyruvate kinase. TC formation was completed by the addition of [^14^C]Phe-tRNA^Phe^ and continued incubation for 2 min at 37 °C. PRE complexes were formed by mixing IC with a 2-fold excess of TC containing [^14^C]Phe-tRNA^Phe^ and purified by centrifugation through 1.1 M sucrose cushion in buffer A containing 20 mM MgCl_2_. The pellets where resuspended in buffer A supplemented with 20 mM MgCl_2_, flash-frozen and stored in small aliquots and diluted to buffer A immediately before use. Efficiency of tRNA incorporation was assessed by nitrocellulose filtration and radioactivity counting.

### Stopped-flow measurements and data fitting

Rapid kinetic experiments were carried out in buffer A at 37 °C. Alexa 488 and fluorescein fluorophores were excited at 465 nm and the emission was recorded after passing through a KV500 cut-off filter (Schott). When FRET between Alexa 488 and Alexa 568 was monitored, the emission of the acceptor fluorophore was recorded after passing a KV590 filter. EF-G-induced translocation was monitored after mixing PRE complexes (0.05 µM) with saturating concentration of EF-G (4–5 µM) in the presence of GTP (1 mM) and where indicated FA (200 μM). All time courses shown represent the average of eight technical replicates. Errors and error bars are s.e.m.. Numerical integration analysis was performed with KinTek Explorer^34, 35^.
